# A causal link or coincidence? Anti-nephrin antibody-positive minimal change disease coexisting with neuroblastoma in a child

**DOI:** 10.1186/s12882-026-04995-x

**Published:** 2026-04-21

**Authors:** Han Li, Sanlong Zhao, Ruochen Che

**Affiliations:** 1 Department of Emergency/Critical Medicine, Children’s Hospital of Nanjing Medical University, 72 Guangzhou Road, Nanjing, Jiangsu Province 210008 China; 2 Department of Nephrology, Children’s Hospital of Nanjing Medical University, 72 Guangzhou Road, Nanjing, Jiangsu Province 210008 China; 3 Nanjing Children’s Hospital, Clinical Teaching Hospital of Medical School, Nanjing University, Nanjing, China

**Keywords:** Neuroblastoma, Minimal change disease, Pediatric case, Anti-nephrin antibody, Case report

## Abstract

**Background:**

The combination of minimal change disease and solid tumors is uncommon, and its mechanism is often associated with immune disorders or metabolic abnormalities.

**Case presentation:**

Here, we describe an 8-year-old girl who presented with nephrotic syndrome characterized by edema and proteinuria as the initial symptoms. Chest imaging incidentally revealed the presence of a chest mass. Subsequently, a PET/CT scan ruled out the possibility of renal infiltration. Following surgical resection of the mass, pathological examination ultimately confirmed it as neuroblastoma. However, even after complete tumor resection, the nephrotic syndrome in the child did not show any relief. A kidney biopsy confirmed minimal change disease, and serum anti-nephrin antibodies were detected. After treatment with glucocorticoids, significant relief of edema, proteinuria, and hypoalbuminemia was observed in the short term.

**Conclusion:**

We report a case of newly diagnosed MCD associated with neuroblastoma. This rare case highlights the importance of integrating clinical manifestations, imaging, pathology, and anti-nephrin antibody testing to assist in diagnosis and treatment.

## Background

Nephrotic syndrome (NS) is a disease process characterized by massive proteinuria and hypoproteinemia due to impaired function of the glomerular filtration barrier [1, 2]. NS has diverse causes, in addition to the primary causes such as minimal change disease(MCD), and some tumors can also cause secondary NS [3]. Common types of tumor include lung cancer, gastric cancer, breast cancer, neuroblastoma, etc [4–6].

Neuroblastoma is a common solid tumor in children that originates from the sympathetic nervous system and commonly occurs in the adrenal medulla or retroperitoneum [7]. As a malignant tumor, the tumor cells release some bioactive substances and stimulate the body’s immune response, thereby inducing systemic symptoms [8, 9]. Up to now, there have been approximately 6 cases of children with neuroblastoma combined with NS, while only 2 cases have specifically described the pathological types of the children’s kidneys, including IgA nephropathy and membranous nephropathy [10, 11]. It is reported that NS associated with neuroblastoma may be related to the deposition of tumor-related immune complexes, but the specific mechanism may be more complex [10, 12].

In this case report, we present a case of newly diagnosed MCD associated with neuroblastoma, presenting with edema and proteinuria as the initial symptoms. Furthermore, we provide a comprehensive literature review of pediatric neuroblastoma-associated nephrotic syndrome and discuss the potential pathogenic role of anti-nephrin antibodies, aiming to offer new insights into the immunological triggers of this rare clinical association.

## Case presentation

**Fig. 1:**
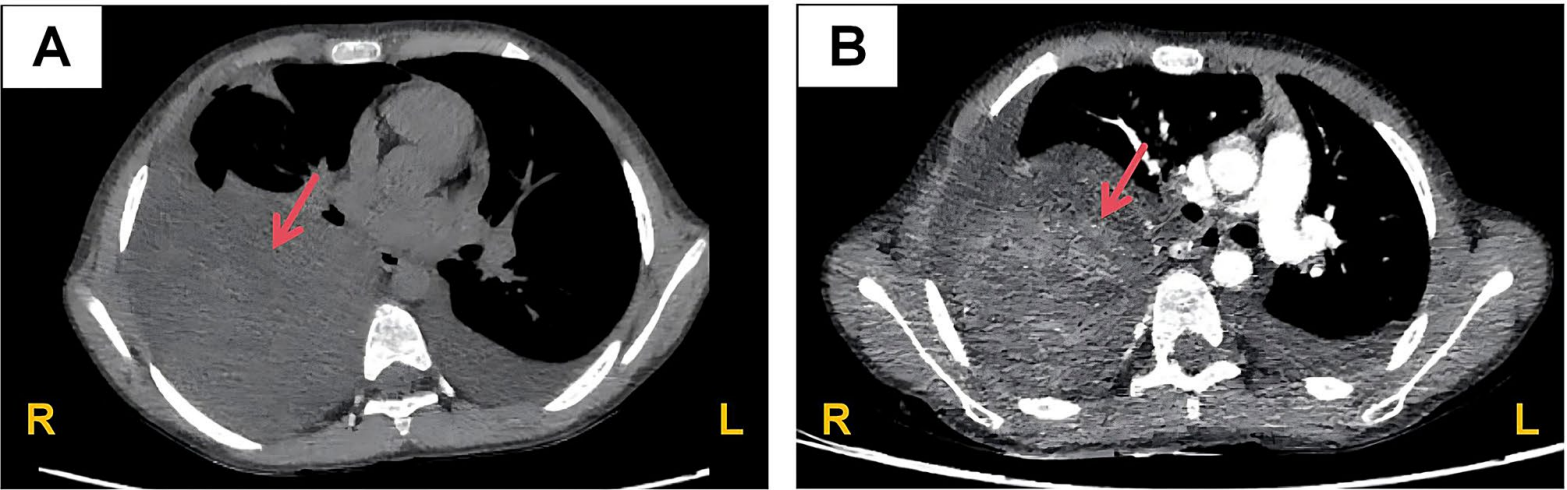
Computerized tomography (CT) images of the child. (**A**) Representative images of chest CT scan; (**B**) Representative images of chest enhanced CT scans

An 8-year-old girl was admitted to the hospital in June 2025 due to generalized edema. She had no history of similar illnesses, exposure to infectious diseases, or autoimmune diseases and no recent history of infection. Her parents were not closely related. Upon admission, she presented with massive proteinuria (urine protein 3+, urine protein-to-creatinine ratio 2.1 mg/mg), hypoalbuminemia (albumin 15.7 g/L), hyperlipidemia (cholesterol 10.09 mmol/L), no hematuria, no hypertension, normal complement levels, and normal kidney function, leading to a diagnosis of primary NS. Unexpectedly, during the physical examination of the child, the right lung had low breath sounds, but the child had no breathing difficulty. Therefore, chest CT and contrast-enhanced CT demonstrated a large, quasi-circular mass located in the right paraspinal region of the posterior mediastinum. The lesion exhibited ill-defined margins and heterogeneous internal density. Concurrent findings included inflammatory changes in the right lung and bilateral pleural effusions (Fig. 1). For further evaluation, serum neuron-specific enolase (NSE) was measured and the level was 19.10 ng/mL. Bone marrow aspiration and cytomorphological analysis showed normocellular bone marrow with active hematopoiesis, and scattered and small clusters of platelets were identified.

**Fig. 2:**
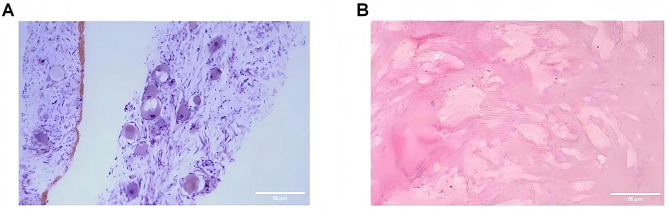
The histopathology of the tumor in the child's chest cavity. (**A**) Representative image of periodic acid-Schiff staining of the tissue from the thoracic mass puncture, 400×, Scale bar = 50 μm; (**B**) Representative image of hematoxylin and eosin staining of the tissue from the thoracic mass resection, 400×, Scale bar = 50 μm

Given the above, the patient was referred to the cardiothoracic surgery department for puncture biopsy of the lesion, and the pathological diagnosis was “neuroblastoma; sent for examination as a ganglioneuroblastoma component,” suggesting neuroblastoma (Fig. 2A). Interestingly, two days after the enhanced CT scan, an abdominal CT scan revealed “uneven density in both kidneys, with increased density shadows within (contrast agent residue)”. This finding indicates that there is a greater possibility of transient contrast agent retention in the renal parenchyma, but renal lesions caused by tumors should also not be ruled out.

To clarify whether the kidney was invaded by the tumor, a PET/CT scan was performed, which showed positive FDG- and SSTR-targeted imaging of the thoracic lesion, with no obvious signs of metastasis and no infiltration of the kidneys. Following a multidisciplinary team (MDT) consultation involving nephrologists, radiologists, oncologists, and thoracic surgeons, a consensus was reached to proceed with the surgical resection of the mediastinal lesion. the postoperative pathology indicated that “the sent tumor tissue was mostly composed of Schwannian matrix and ganglion cells of different degrees of differentiation (approximately 90%), with a small amount of scattered focal differentiated neuroblastoma components containing a neural plexiform background (approximately 10%); focal lymphocyte infiltration and calcification were observed in the tumor tissue”, which was analyzed as ganglioneuroblastoma (mixed type). Immunohistochemical staining revealed positivity for the PHOX2B, Ki67 and Syn proteins (Fig. 2B). Moreover, fluorescence in situ hybridization did not reveal N-MYC amplification. After evaluation by the oncology department, the tumor was classified as low-risk, and no adjuvant therapy (chemotherapy or radiotherapy) was recommended.

**Fig. 3:**
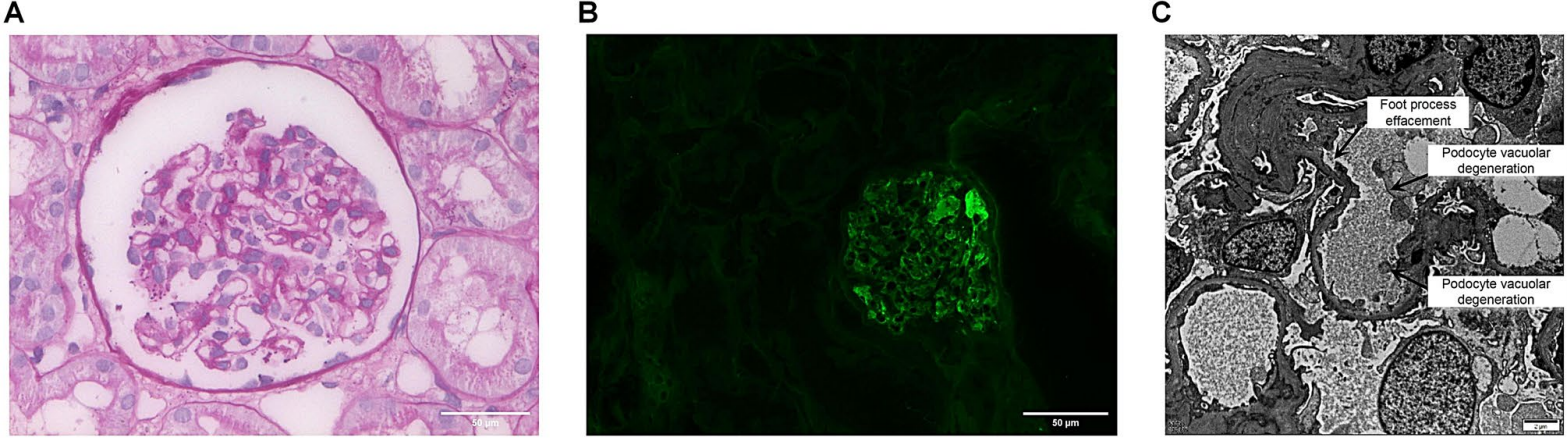
Pathological examination of the renal biopsy. (**A**) Light microscopy showing a relatively normal-appearing glomerulus with podocyte swelling (PAS staining, 400×; scale bar = 50 μm). (**B**) Immunofluorescence showing segmental granular IgM deposition along the GBM and mesangium, whereas IgG was negative (400×; scale bar = 50 μm; Green, IgM). (**C**) Electron microscopy highlighting diffuse foot process effacement and podocyte vacuolar degeneration (4000×; scale bar = 2 μm)

The child’s edema improved and urine output increased within two weeks after surgery; however, at nearly one month postoperatively, the patient still exhibited massive proteinuria and hypoproteinemia, with no significant improvement compared to preoperative levels. Further testing for serum anti-nephrin antibody was performed using the second-generation high-sensitivity Super Nephrin AntibodyTrap (SuperNAT) assay, an independently developed method by Aogen Diagnostics [13, 14]. The assay result was 130.01 U/mL, with a normal reference range of < 45 U/mL, indicating a positive expression of anti-nephrin antibody. As a marker associated with renal podocyte injury, the presence of anti-nephrin antibody suggests immune-mediated podocyte damage, which may contribute to the pathogenesis of the renal lesions observed in this patient. But its precise role in this specific case remains speculative given the lack of dynamic monitoring. Subsequently, ultrasound-guided renal biopsy was performed in the patient. Light microscopy showed podocyte swelling without mesangial proliferation, crescent formation, or basement membrane remodeling (e.g., spikes or tram-track changes). Immunofluorescence demonstrated segmental granular IgM deposition (+) within the mesangium and along the glomerular basement membrane (GBM), while IgG, IgA, C1q, C3, and fibrinogen were consistently negative. Electron microscopy further revealed diffuse foot process effacement (> 80%), microvillous transformation, and vacuolar degeneration of podocytes, with no definite electron-dense deposits. These findings were consistent with a diagnosis of MCD (Fig. 3). Therefore, the child received standard initial induction therapy with prednisone at 2 mg/kg/day (consistent with recommendations from the ISN/IPNA and KDIGO guidelines) for the first month. Following treatment, edema, proteinuria, and hypoproteinemia were significantly relieved within two weeks and completely resolved at 1 month. Subsequently, the prednisone dose was gradually tapered in accordance with the above guidelines. No relapse or tumor recurrence was observed during a follow-up period of 5 months.

## Discussion and conclusion

Cases of childhood tumors accompanied by NS are rarely reported. Neuroblastoma is a malignant tumor specific to children [15]. However, in cases of NS combined with childhood tumors, neuroblastoma accounts for an extremely low proportion of cases, which is far lower than the association frequency of lymphoma, leukemia, and other tumors with NS [16, 17]. In pediatric cases, only one case of nephrotic syndrome presenting as immune glomerulonephritis combined with neuroblastoma was reported in 1979, and another case of IgA nephropathy combined with neuroblastoma was reported in 2024 [10, 11]. Additionally, there is another case of a 10-month-old female infant with neuroblastoma presenting as acute kidney injury, hyponatremia-hypertensive-like syndrome, and nephrotic proteinuria. This case lacks pathological data from kidney biopsy, but considering the clinical manifestations and other factors, it does not support a diagnosis of MCD [18]. Therefore, as far as we know, in cases of neuroblastoma combined with NS, this study documents a rare instance specifically characterized by the pathological type of minimal change.

To explore the potential clinical association between neuroblastoma and MCD, three potential mechanisms have been systematically evaluated: (1) a true paraneoplastic syndrome; (2) coincidental coexistence of two independent rare conditions; (3) primary NS unmasked or exacerbated by the malignancy.

First, regarding the potential for a paraneoplastic syndrome, some evidence indicates that MCD represents a paraneoplastic renal syndrome secondary to the tumor [19]. Paraneoplastic renal syndrome refers to renal dysfunction indirectly caused by the abnormal immune response of the body to tumor cells or the bioactive substances secreted by the tumor rather than renal metastasis of the tumor [20]. The renal histologic and ultrastructural findings in this patient were distinct from those of neuroblastoma and consistent with MCD. Electron microscopy showed only diffuse podocyte injury with foot process effacement and microvillous transformation, lacking neurosecretory granules, neural processes, or malignant cellular features typical of neuroblastic tumors. This clearly distinguishes primary podocytopathy from direct renal infiltration by neuroblastoma. Meanwhile, the occurrence of MCD in the context of neuroblastoma supports a paraneoplastic association rather than direct tumor invasion. The pathogenesis may involve tumor-derived cytokines or circulating factors mediating podocyte injury, raising the possibility of a paraneoplastic association rather than direct tumor invasion.

In a typical paraneoplastic process, a causal relationship would be supported by resolution or improvement of NS after tumor resection, or exacerbation of NS with tumor progression [21]. However, in this case, there was no clear temporal sequence of occurrence between the child’s NS and neuroblastoma. Interestingly, although significant proteinuria persisted for nearly one month after tumor resection, the patient experienced a noticeable improvement in edema and an increase in urine output during the immediate postoperative period. These clinical changes, while partial, may suggest a potential paraneoplastic component where the tumor contributed to the severity of the renal manifestations. Of note, emerging evidence has demonstrated that in some paraneoplastic glomerulopathies, the systemic immune cascade may remain activated even after complete tumor resection, and additional immunosuppressive treatment such as corticosteroids is often required to achieve remission of proteinuria [22]. This suggests that the MCD might not be a classical paraneoplastic syndrome directly driven by tumor-secreted factors. Instead, the neuroblastoma may have acted as an immunological trigger that disrupted immune tolerance, or the two conditions might have co-existed independently. Therefore, while the persistent proteinuria makes a purely paraneoplastic etiology less certain, a mixed mechanism or the unmasking of an underlying primary NS by tumor-related inflammation cannot be excluded.

Second, the possibility of coincidental coexistence is equally noteworthy. It is possible that neuroblastoma, particularly biologically favorable types or those differentiating toward ganglioneuroblastoma, can be detected incidentally during the diagnostic workup for unrelated symptoms like NS. Given the rarity of their association, the possibility of an incidental finding must be carefully considered.

Third, another equally important possibility involves primary NS being unmasked or exacerbated by the malignancy. The presence of anti-nephrin antibodies characterizes the immunological features of the podocytopathy in this patient. Nephrin is a key protein on the slit membrane of glomerular podocytes that plays a crucial role in maintaining the integrity of the glomerular filtration barrier [23]. Anti-nephrin antibodies can attack nephrin on the surface of podocytes through an immune response, leading to the destruction or dysfunction of nephrin structure, and subsequently damaging the slit diaphragm [24]. Anti-nephrin antibodies may contribute to podocyte injury in primary NS, whereas secondary NS, including tumor-associated cases, is typically mediated by immune complex deposition or metabolic factors [25, 26]. However, to maintain a balanced interpretation, we must consider that the pathophysiology of paraneoplastic NS is complex, involving cytokine dysregulation, T-cell–mediated responses, and the induction of autoantibodies through molecular mimicry [27]. In this scenario, one could speculate whether the neuroblastoma acted as an “immunological trigger” that broke immune tolerance, leading to the production of autoantibodies in a predisposed patient [13]. Consequently, a dualistic perspective offers a possible framework for interpreting these observations: the patient might have had an underlying primary podocytopathy that was either incidentally discovered or significantly exacerbated by the neuroblastoma. This mixed etiology explains the partial clinical response to surgery and the subsequent requirement for glucocorticoids to achieve full remission. In conclusion, neuroblastoma combined with MCD is extremely rare. Here, we present a unique case of a pediatric patient with the coexistence of neuroblastoma and MCD. Following surgical resection of the tumor, the child still had significant proteinuria and hypoalbuminemia. PET/CT and kidney biopsy ruled out the possibility of tumor invasion in kidney. After treatment with glucocorticoids, significant relief of edema, proteinuria, and hypoalbuminemia was observed within a short period.

Of course, in this case, there are still some limitations that need to be noted. First, MIBG scintigraphy, which is a standard functional imaging modality for neuroblastoma, was not performed in this patient, which represents a limitation of this report. Second, we lacked immunofluorescence evidence demonstrating antibody deposition in the kidney tissue, which means that we cannot definitively confirm that the circulating antibodies were the direct cause of the podocyte effacement observed. Moreover, the study lacked pre- and post-intervention anti-nephrin antibody monitoring. Without these data, we cannot establish a clear temporal correlation between the tumor burden, antibody titers, and clinical remission. Finally, the interpretation of the relationship between the tumor and NS is based on clinical speculation and lacks sufficient evidence for confirmation. While the presence of anti-nephrin antibodies defines the immunological phenotype of the NS, it remains difficult to elucidate whether the neuroblastoma was a primary driver through paraneoplastic mechanisms, a coincidental incidentaloma, or part of a mixed etiology where the malignancy triggered an underlying immune predisposition. Nevertheless, this case enriches the clinical understanding of the rare coexistence of neuroblastoma and NS, and emphasizes the importance of a balanced etiological evaluation and the necessity of conducting a comprehensive evaluation for anti-nephrin antibodies in similar clinical scenarios.

## Data Availability

The datasets generated during and/or analysed during the current study are available from the corresponding author on reasonable request.

## Abbreviations

MCD: Minimal change disease
NS: Nephrotic syndrome
NSE: Neuron-specific enolase
MDT: Multidisciplinary team
